# Association of the single point insulin sensitivity estimator index with functional outcomes and hemorrhagic transformation in patients with acute ischemic stroke

**DOI:** 10.3389/fnut.2026.1805357

**Published:** 2026-06-22

**Authors:** Zicheng Cheng, Xinxia Zhu, Lingfan Xia, Yifeng Wu, Fangwang Fu, Zhao Han, Tong Xu

**Affiliations:** 1 Department of Neurology, The Second Affiliated Hospital and Yuying Children's Hospital of Wenzhou Medical University, Wenzhou, China; 2 Department of Neurology, Taizhou First People's Hospital, Taizhou, China

**Keywords:** acute ischemic stroke, insulin resistance, predictive performance, prognosis, single point insulin sensitivity estimator

## Abstract

**Background:**

Insulin resistance is an important risk factor for adverse prognosis in acute ischemic stroke (AIS). The single point insulin sensitivity estimator (SPISE) index, a novel insulin resistance surrogate independent of fasting plasma glucose, offers a potential advantage for assessing insulin resistance in AIS due to its lower susceptibility to stress-induced hyperglycemia in the acute phase. However, its associations with functional outcomes and intracranial hemorrhagic transformation (HT) in AIS remain unclear.

**Methods:**

This multicenter retrospective cohort study consecutively enrolled patients with AIS who received intravenous thrombolysis between January 2019 and December 2023. We calculated five insulin resistance surrogates (SPISE index, TyG index, TyG-BMI, TG/HDL-C ratio, and METS-IR) and employed multivariable logistic regression models to analyze their associations with an unfavorable 90-day functional outcome (modified Rankin Scale score 3–6) and post-thrombolysis HT. The predictive performance of each index was compared using the area under the receiver operating characteristic curve (AUC).

**Results:**

Among the 862 patients ultimately included, the levels of the SPISE index, TyG index, TyG-BMI, and METS-IR were all independently associated with the risk of unfavorable functional outcomes (all *P* < 0.05). Specifically, the SPISE index showed a linear inverse association with this risk [odds ratio (OR) 0.83, 95% confidence interval (CI) 0.74–0.92, *P* for nonlinearity = 0.83]. Regarding predictive performance, METS-IR showed the highest discriminatory ability (AUC = 0.580), followed by TyG-BMI (AUC = 0.576). The SPISE index (AUC = 0.557) and the TyG index (AUC = 0.555) demonstrated comparable performance. For HT, only the TG/HDL-C ratio was independently associated with an increased risk (OR 1.09, 95% CI 1.01–1.18), while no significant associations were observed for the other surrogate indices.

**Conclusions:**

In patients with AIS receiving intravenous thrombolysis, several insulin resistance surrogates are independently associated with unfavorable functional outcomes. However, all surrogate indices showed limited standalone discrimination, indicating they are not suitable as individual prognostic tools in clinical practice.

## Introduction

Stroke ranks as the second leading contributor to the global burden of non-communicable diseases (1). In China, ischemic stroke accounts for more than 80% of all stroke cases, and its treatment is highly time-dependent (2). Studies indicate that for each minute of cerebral blood flow interruption, an average of 1.9 million neurons and 14 billion synapses are lost (3). Consequently, the cornerstone of acute ischemic stroke (AIS) treatment lies in achieving timely vascular recanalization, such as through intravenous thrombolysis, to salvage the ischemic penumbra (4). Despite receiving intravenous thrombolysis, approximately one-third of patients remain functionally dependent at 3 months after onset (5). An additional therapeutic concern is the risk of intracranial hemorrhagic transformation (HT) associated with intravenous thrombolysis, which occurs in about 10% of cases (5). This not only may directly lead to neurological deterioration but also delays crucial secondary preventive antithrombotic therapy, ultimately associated with unfavorable functional outcomes and an increased risk of post-stroke epilepsy (6, 7). Therefore, identifying risk factors for unfavorable functional outcomes and HT in patients undergoing intravenous thrombolysis is essential for guiding individualized treatment strategies and optimizing risk management.

Insulin resistance refers to a decreased sensitivity or responsiveness of tissues to insulin (8). Insulin resistance drives the progression of atherosclerosis and thrombus formation by inducing low-grade chronic inflammation, oxidative stress, dyslipidemia, endothelial dysfunction, and a hypercoagulable state, thereby increasing the risk of cardiovascular and cerebrovascular events (9). In clinical research, the hyperinsulinemic-euglycemic clamp (HEC), the gold standard for assessing insulin resistance, is difficult to apply in patients with AIS due to its complex operation, high cost, and invasive nature (10). Consequently, a series of surrogate indices based on routine blood tests and physical examination have emerged and are primarily divided into two categories: (1) indices dependent on insulin measurement, such as the homeostasis model assessment of insulin resistance (HOMA-IR) and the quantitative insulin sensitivity check index (QUICKI) (10); and (2) indices independent of insulin measurement, such as the triglyceride-glucose (TyG) index (11), the TyG-body mass index (TyG-BMI) (12), the triglyceride to high-density lipoprotein cholesterol (TG/HDL-C) ratio (13), and the metabolic score for insulin resistance (METS-IR) (14).

In the field of AIS, HOMA-IR has been demonstrated to be associated with unfavorable functional outcomes in patients (15, 16). However, because insulin measurement is not a routine test in the acute phase, especially for non-diabetic patients, its clinical utility is limited. As a result, research focus has shifted toward insulin-independent surrogate indices. Recent studies have confirmed that the TyG index (17), TyG-BMI (18, 19), and METS-IR (20, 21) are independently associated with functional outcomes in patients with AIS and demonstrate good predictive performance. It is important to note, however, that all three surrogate indices incorporate fasting plasma glucose (FPG) as a calculation parameter. As is widely recognized, FPG often exhibits reactive elevation under the stress of AIS (22). This may lead to significant fluctuations in the assessed level of insulin resistance based on these surrogate indices around the time of stroke onset. The single point insulin sensitivity estimator (SPISE) index, a novel insulin resistance surrogate, is calculated based on TG, HDL-C, and BMI, and shows a good correlation with insulin resistance measured by the HEC (23). Crucially, the SPISE index does not rely on FBG measurement, making it theoretically less susceptible to the influence of stress-induced hyperglycemia in the acute phase of AIS. We therefore hypothesize that, compared to surrogate indices incorporating FBG, the SPISE index may provide a more stable and specific tool for assessing insulin resistance in patients with AIS, potentially offering superior prognostic predictive ability.

However, no studies to date have evaluated the relationship between the SPISE index and functional outcomes in AIS. Furthermore, evidence regarding the association between insulin resistance and the critical complication of post-thrombolysis HT is scarce (24–27), which is disproportionate to the importance of this issue. Thus, this study aimed to investigate the associations of the SPISE index with 3-month unfavorable functional outcomes and post-thrombolysis HT in patients with AIS receiving intravenous thrombolysis. Additionally, it aimed to compare the predictive performance of the SPISE index with that of other insulin resistance surrogates for the aforementioned clinical endpoints, in order to test our hypothesis.

## Methods

### Patient selection

This study was a multicenter retrospective cohort study, conducted in accordance with the Strengthening the Reporting of Observational Studies in Epidemiology (STROBE) statement guidelines (28). We retrospectively and consecutively enrolled patients with AIS who were admitted to the Second Affiliated Hospital of Wenzhou Medical University and Taizhou First People's Hospital between January 2019 and December 2023. The patient screening flow chart is presented in Supplementary Figure 1. Eligibility for initial screening required meeting the following inclusion criteria: (1) a diagnosis of AIS confirmed by both clinical assessment and neuroimaging; (2) receipt of intravenous thrombolysis with alteplase or tenecteplase within 4.5 h of symptom onset; (3) age over 18 years; and (4) availability of complete baseline clinical data, treatment records, and imaging data. Based on these criteria, 1,170 patients were initially screened. Exclusion criteria were: (1) discontinuation of intravenous thrombolysis for reasons other than HT (*n* = 10); (2) missing baseline data required for calculating the SPISE index (*n* = 159); 3) lack of 90-day functional outcome data assessed by the modified Rankin Scale (mRS) (*n* = 83); (4) presence of other comorbid conditions that significantly impair activities of daily living, such as dementia, Parkinson's disease, severe cardiac insufficiency, or major fractures (*n* = 21); and (5) missing key treatment variables related to intravenous thrombolysis, such as the baseline National Institutes of Health Stroke Scale (NIHSS) score, thrombolytic drug dose, or onset-to-treatment time (OTT) (*n* = 35). Finally, 862 patients were included in the analysis. The study protocol was approved by the Ethics Committees of the Second Affiliated Hospital of Wenzhou Medical University and Taizhou First People's Hospital, and the requirement for informed consent was waived. The study was conducted in full accordance with the ethical principles outlined in the Declaration of Helsinki.

### Data collection and variable definitions

Baseline demographic, clinical, and laboratory data were retrospectively collected from the electronic medical record systems of the two participating centers. To ensure data accuracy, extraction and entry were performed independently by two trained researchers, with subsequent cross-verification. The collected variables included age, sex, and vascular risk factors (including hypertension, diabetes mellitus, hyperlipidemia, atrial fibrillation, coronary heart disease, history of stroke, and current smoking). Medication history prior to admission, initial stroke severity as assessed by the NIHSS score, blood pressure on admission, and BMI were also recorded. Information regarding intravenous thrombolysis encompassed the type of thrombolytic agent (alteplase or tenecteplase), the specific dose administered, the OTT, and whether bridging endovascular therapy was performed. Laboratory parameters analyzed were obtained from fasting blood samples drawn on the morning following admission. These included FPG, glycated hemoglobin (HbA1c), a full lipid profile [total cholesterol (TC), low-density lipoprotein cholesterol (LDL-C), HDL-C, and TG], and serum creatinine. The estimated glomerular filtration rate (eGFR) was calculated using the 2021 Chronic Kidney Disease Epidemiology Collaboration (CKD-EPI) creatinine equation. Results from standard auxiliary examinations, such as electrocardiography, transthoracic echocardiography, cranial computed tomography (CT), magnetic resonance imaging (MRI), and CT or MR angiography of the head and neck, were reviewed.

The definition criteria for some variables are as follows. Hypertension was defined as a prior diagnosis, current use of antihypertensive medication, or systolic/diastolic blood pressure ≥140/90 mmHg on three separate measurements after admission. Diabetes mellitus was defined by a prior diagnosis, use of antidiabetic agents, or an HbA1c level ≥6.5%. Hyperlipidemia was defined as a prior diagnosis, use of lipid-lowering therapy, or a TC level ≥200 mg/dL or LDL-C ≥130 mg/dL. Regarding intravenous thrombolysis, the standard doses were defined as 0.9 mg/kg (max 90 mg) for alteplase and 0.25 mg/kg (max 25 mg) for tenecteplase; administration below these thresholds was categorized as a low dose. Infarct location (anterior circulation, posterior circulation, or both) was determined based on CT/MRI findings. Stroke etiology was classified according to the Trial of Org 10,172 in Acute Stroke Treatment (TOAST) criteria (29) by two neurologists blinded to the outcomes, with a third senior neurologist serving as an adjudicator in case of disagreement.

### Insulin resistance surrogates

Five surrogate indices of insulin resistance (the SPISE index, TyG index, TyG-BMI, TG/HDL-C ratio, and METS-IR) were calculated based on FBG, lipid profile, and BMI data obtained after patient admission. The formulas used for calculation are as follows:

SPISE index = (600 × HDL-C (mg/dL)^0.185^) / (TG (mg/dL)^0.2^ ×
BMI (kg/m^2^)^1.338^)
TyG index = Ln [TG (mg/dL) × FPG (mg/dL) / 2]
TyG-BMI = TyG index × BMI (kg/m^2^)
TG/HDL-C ratio = TG (mg/dL) / HDL-C (mg/dL)
METS-IR = Ln [(2 × FPG (mg/dL) + TG (mg/dL)] × BMI
(kg/m^2^) / Ln [HDL-C (mg/dL)]

### Outcomes

The primary outcome of this study was functional outcomes at 90 days after stroke onset, assessed using the mRS. Evaluations were conducted via standardized telephone follow-up or outpatient review by trained researchers who were blinded to the patients' laboratory parameters. Functional outcomes were dichotomized as functional independence (mRS score 0–2) and unfavorable functional outcome (mRS score 3–6) (30). The secondary outcome was post-thrombolysis HT, defined according to the National Institute of Neurological Disorders and Stroke (NINDS) criteria as any new hemorrhage detected on follow-up cranial CT performed 24–36 hours after intravenous thrombolysis (31).

### Statistical analysis

All statistical analyses were performed using R software (version 4.4.2; R Foundation for Statistical Computing, Vienna, Austria). A two-sided *P* value < 0.05 was considered statistically significant. Patient characteristics were described according to the functional outcome groups. Continuous variables are presented as mean ± standard deviation (SD) or median (25th−75th percentile), and were compared using the Student's *t*-test or Mann-Whitney U test, as appropriate. Categorical variables are presented as number (percentage) and were compared using the Chi-square test or Fisher's exact test. Pairwise correlations among the five insulin resistance surrogates were assessed using Spearman's rank correlation analysis. Binary logistic regression models were employed to evaluate the association between each insulin resistance surrogate and the unfavorable functional outcome. Given the high pairwise correlations among insulin resistance surrogates (e.g., ρ=-0.97 between SPISE and TyG-BMI), we did not include multiple surrogate indices in the same regression model to avoid multicollinearity bias. Instead, each surrogate index was analyzed separately to estimate its independent association with outcomes. The SPISE index was included in the models in two forms: as a continuous variable and as a categorical variable divided by sample quartiles. Three levels of models were constructed: (1) a crude Model, in which each insulin resistance surrogate was entered separately; (2) Model 1, adjusted for all variables that showed an association with the outcome at *P* < 0.1 in the univariate analysis; and (3) Model 2, which was further adjusted for pre-specified confounding factors based on clinical knowledge and literature, in addition to the variables in model 1. Before constructing the multivariate models, multicollinearity among independent variables was assessed using the variance inflation factor (VIF). A VIF ≥10 indicated severe collinearity, and the variable was handled accordingly. To evaluate the heterogeneity of the associations, subgroup analyses were performed according to the following pre-specified stratifying variables: age ( ≤ 60 years/>60 years), sex (female/male), BMI (< 24/≥24 kg/m^2^), history of diabetes mellitus (no/yes), and baseline NIHSS score ( ≤ 5/>5). The association between the SPISE index and unfavorable functional outcomes was analyzed within each subgroup. Restricted cubic spline (RCS) regression models, adjusted for all covariates in Model 2, were used to explore a potential non-linear dose-response relationship between the SPISE index and unfavorable functional outcomes. Three knots were set in the RCS regression model (32), with the 25th percentile as the reference value. The discriminatory ability of each insulin resistance surrogate for unfavorable functional outcomes was evaluated using receiver operating characteristic (ROC) curves, and the area under the curve (AUC) was calculated. The DeLong test was used to compare the statistical differences in AUCs between different surrogate indices.

## Results

### Patient characteristics

This study ultimately included 862 patients with AIS. The mean age of the cohort was 68.1 ± 13.2 years, and 585 patients (67.9%) were male. The median baseline NIHSS score was 4.0 [interquartile range (IQR) 3.0–9.0]. The vast majority of patients (91.3%) received standard-dose intravenous thrombolysis, and 46 patients (5.3%) subsequently underwent bridging endovascular therapy. At 90 days post-stroke, 663 patients (76.9%) achieved functional independence, while 199 patients (23.1%) had an unfavorable functional outcome. As shown in Table 1, compared to the functional independence group, patients in the unfavorable functional outcome group were older and had higher baseline NIHSS scores, systolic blood pressure, FBG, HbA1c, and BMI but a lower eGFR. This group also had a higher prevalence of hypertension and atrial fibrillation, and a higher proportion received bridging therapy. Regarding etiological distribution, the unfavorable functional outcome group had a higher proportion of large artery atherosclerosis and cardioembolic strokes compared to small vessel occlusion strokes. Overall, HT occurred in 76 patients (8.8%). A comparison of baseline characteristics between patients with and without HT is presented in Supplementary Table 1.

**Table 1:** Characteristics of patients stratified by functional outcomes.

Characteristic	Overall (*N* = 862)	Functional independence (*N* = 663)	Unfavorable functional outcomes (*N* = 199)	*P*-value
Age, years, mean ± SD	68.1 ± 13.2	67.2 ± 12.9	71.1 ± 13.7	< 0.001
Sex, *n* (%)				
Female	277 (32.1)	203 (30.6)	74 (37.2)	0.082
Male	585 (67.9)	460 (69.4)	125 (62.8)	
Hypertension, *n* (%)	681 (79.0)	510 (76.9)	171 (85.9)	0.006
Diabetes mellitus, *n* (%)	228 (26.5)	168 (25.3)	60 (30.2)	0.18
Hyperlipidemia, *n* (%)	335 (38.9)	254 (38.4)	81 (40.7)	0.54
Atrial fibrillation, *n* (%)	206 (23.9)	146 (22.0)	60 (30.2)	0.018
Coronary heart disease, *n* (%)	56 (6.5)	39 (5.9)	17 (8.5)	0.18
History of stroke, *n* (%)	144 (16.7)	107 (16.1)	37 (18.6)	0.42
Current smoking, *n* (%)	288 (33.4)	223 (33.6)	65 (32.7)	0.80
Ongoing antithrombotic therapy, *n* (%)	128 (14.8)	91 (13.7)	37 (18.6)	0.090
Baseline NIHSS score, median (Q1–Q3)	4.0 (3.0–9.0)	4.0 (2.0–7.0)	10.0 (5.0–16.0)	< 0.001
Baseline SBP, mmHg, mean ± SD	153.7 ± 22.2	152.8 ± 21.5	156.6 ± 24.1	0.049
Baseline DBP, mmHg, mean ± SD	86.5 ± 14.9	86.3 ± 14.5	87.0 ± 16.2	0.61
BMI, kg/m^2^, mean ± SD	23.6 ± 3.6	23.4 ± 3.6	24.3 ± 3.8	0.004
Thrombolytic drug, *n* (%)				
Alteplase	850 (98.7)	653 (98.6)	197 (99.0)	>0.99
Tenecteplase	11 (1.3)	9 (1.4)	2 (1.0)	
Standard dose, *n* (%)	787 (91.3)	611 (92.2)	176 (88.4)	0.10
OTT, min, median (Q1–Q3)	150.0 (110.0–210.0)	150.0 (110.0–210.0)	150.0 (116.0–210.0)	0.96
Bridge therapy, *n* (%)	46 (5.3)	16 (2.4)	30 (15.1)	< 0.001
eGFR, ml/min/1.73m^2^, median (Q1–Q3)	89.7 (70.7–100.2)	90.9 (73.4–100.6)	85.6 (62.6–97.7)	< 0.001
FPG, mg/dL, median (Q1–Q3)	95 (84–113)	92 (83–108)	104 (91–131)	< 0.001
HbA1c, %, median (Q1–Q3)	5.8 (5.5–6.4)	5.8 (5.5–6.3)	5.9 (5.5–6.7)	0.038
TC, mg/dL, mean ± SD	170.9 ± 42.2	171.9 ± 43.0	167.6 ± 39.5	0.19
LDL-C, mg/dL, mean ± SD	106.0 ± 33.4	106.7 ± 33.5	103.8 ± 33.0	0.27
HDL-C, mg/dL, mean ± SD	43.9 ± 11.0	44.1 ± 11.0	43.5 ± 11.0	0.54
TG, mg/dL, median (Q1–Q3)	115.1 (84.1–163.9)	115.1 (84.1–165.6)	115.1 (83.3–163.0)	0.96
Stroke etiology, *n* (%)				
Large artery atherosclerosis	194 (22.5)	131 (19.8)	63 (31.7)	< 0.001
Cardioembolism	205 (23.8)	149 (22.5)	56 (28.1)	
Small vessel occlusion	248 (28.8)	217 (32.7)	31 (15.6)	
Other determined or undetermined	215 (24.9)	166 (25.0)	49 (24.6)	
Infarct location, *n* (%)				
Anterior circulation	641 (74.4)	488 (73.6)	153 (76.9)	0.53
Posterior circulation	185 (21.5)	148 (22.3)	37 (18.6)	
Anterior and posterior circulation	36 (4.2)	27 (4.1)	9 (4.5)	
SPISE index, median (Q1–Q3)	6.8 (5.6–8.2)	6.9 (5.7–8.3)	6.5 (5.4–7.8)	0.015
Quartiles of SPISE index, *n* (%)				
Q1	216 (25.1)	155 (23.4)	61 (30.7)	0.061
Q2	215 (24.9)	161 (24.3)	54 (27.1)	
Q3	215 (24.9)	170 (25.6)	45 (22.6)	
Q4	216 (25.1)	177 (26.7)	39 (19.6)	
TyG index, median (Q1–Q3)	8.6 (8.3–9.1)	8.6 (8.2–9.1)	8.7 (8.3–9.0)	0.019
TyG-BMI, mean ± SD	206.4 ± 39.4	204.1 ± 38.7	214.4 ± 40.7	0.002
TG/HDL-C ratio, median (Q1–Q3)	2.7 (1.8–4.2)	2.7 (1.8–4.2)	2.8 (1.8–4.1)	0.83
METS-IR, median (Q1–Q3)	36.1 (31.8–41.4)	35.6 (31.2–40.6)	38.0 (33.4–43.3)	< 0.001

BMI, body mass index; DBP, diastolic blood pressure; eGFR, estimated glomerular filtration rate; FPG, fasting plasma glucose; HbA1c, glycated hemoglobin; HDL-C, high-density lipoprotein cholesterol; LDL-C, low-density lipoprotein cholesterol; METS-IR, metabolic score for insulin resistance; NIHSS, National Institutes of Health Stroke Scale; OTT, onset-to-treatment time; SBP, systolic blood pressure; SD, standard deviation; SPISE, single point insulin sensitivity estimator; TC, total cholesterol; TG, triglyceride; TyG, triglyceride-glucose.

### Correlations between insulin resistance surrogates

**Figure 1:**
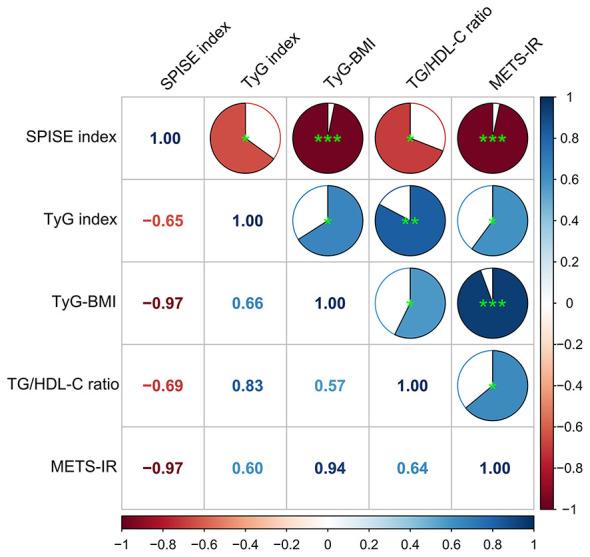
Correlation heatmap displaying the correlation between different insulin resistance surrogates. *P < 0.05, **P < 0.01, ***P < 0.001.

The average levels of the insulin resistance surrogates were as follows: SPISE index, 6.8 (IQR 5.6–8.2); TyG index, 8.6 (IQR 8.3–9.1); TyG-BMI, 206.4 ± 39.4; TG/HDL-C ratio, 2.7 (IQR 1.8–4.2); and METS-IR, 36.1 (IQR 31.8–41.4). Spearman correlation analysis revealed significant pairwise correlations among all five surrogate indices (all *P* < 0.05; Figure 1). Specifically, the SPISE index showed strong negative correlations with TyG-BMI (ρ = −0.97) and METS-IR (ρ = −0.97), and moderate negative correlations with the TyG index (ρ = −0.65) and the TG/HDL-C ratio (ρ = −0.69). The weakest correlation was observed between TyG-BMI and the TG/HDL-C ratio (ρ = 0.57).

### Insulin resistance surrogates and functional outcomes

**Figure 2:**
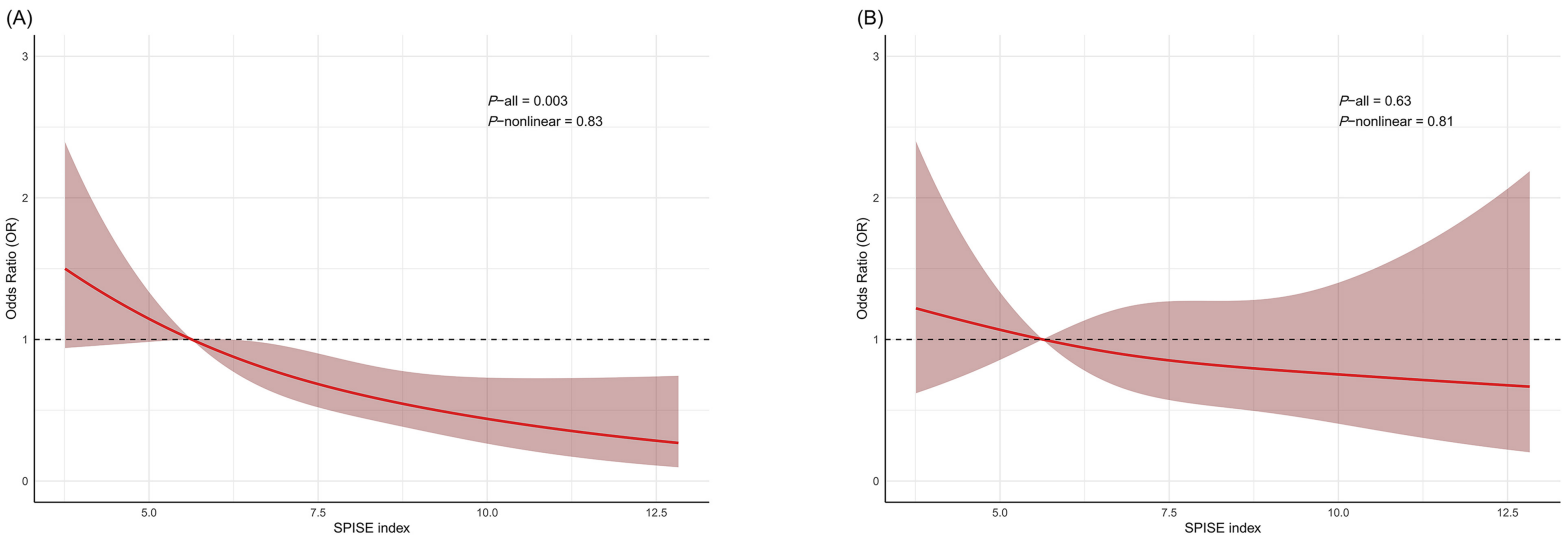
Restricted cubic spline regression model of the association of SPISE index with **(A)** unfavorable functional outcomes and **(B)** hemorrhagic transformation in patients with acute ischemic stroke. CI, confidence interval; OR, odds ratio.

**Figure 3:**
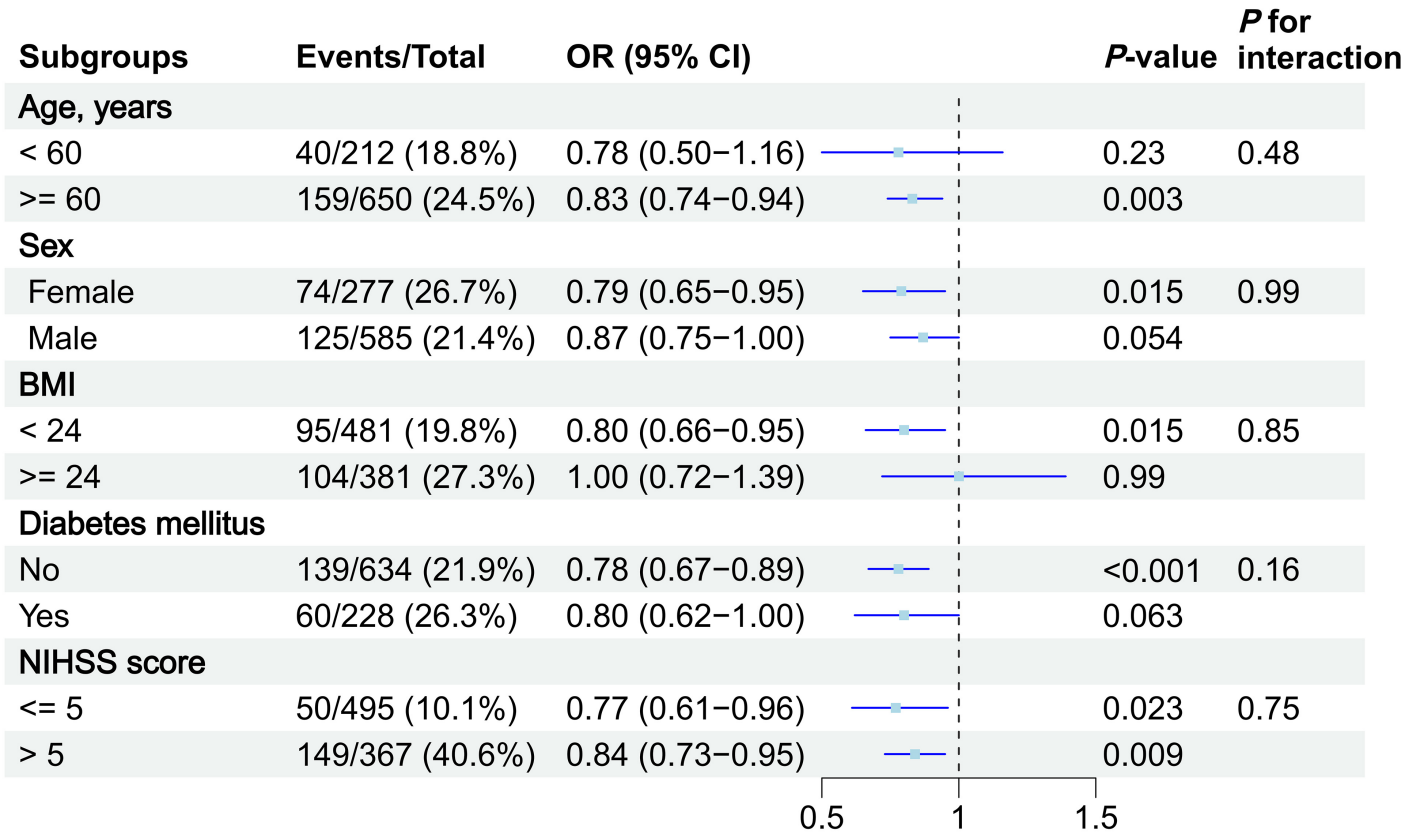
Association between SPISE index and unfavorable outcomes of patients with acute ischemic stroke among different subgroups.

**Figure 4:**
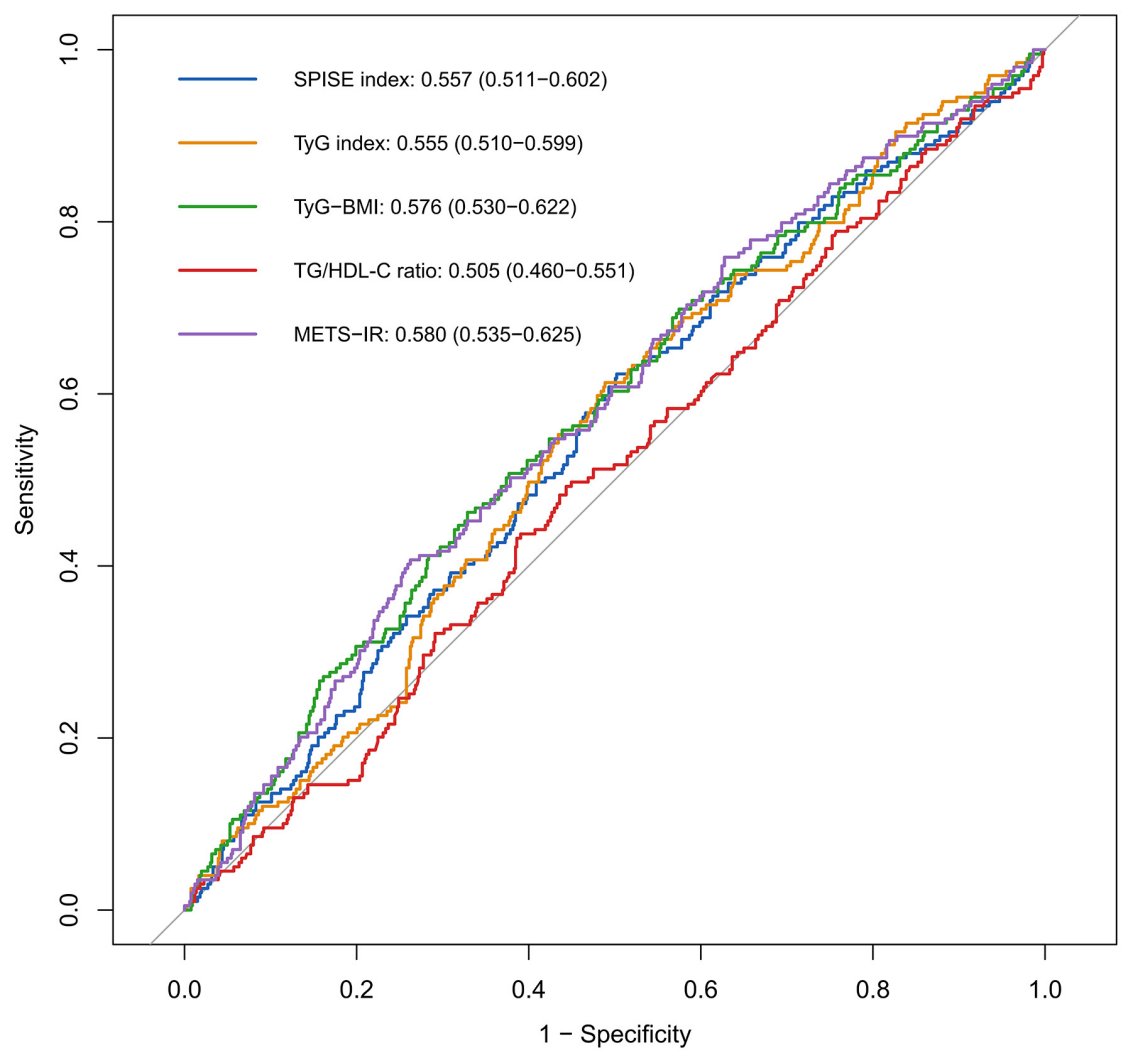
Receiver operating characteristic curves of the effects of different insulin resistance surrogates on the unfavorable functional outcomes in patients with acute ischemic stroke.

Univariate analysis revealed that the unfavorable functional outcome group had a lower SPISE index but higher TyG index, TyG-BMI, and METS-IR compared to the functional independence group (all *P* < 0.05; Table 1). In multivariable logistic regression analysis with full adjustment for confounders (Model 2), each 1-unit increase in the SPISE index was associated with a 17% reduction in the likelihood of unfavorable functional outcomes [odds ratio (OR) 0.83, 95% confidence interval (CI) 0.74–0.92]. When analyzed by quartiles, compared to the highest quartile (Q4), the Q1, Q2, and Q3 groups had a significantly higher likelihood of unfavorable functional outcomes (*P* for trend < 0.001). RCS analysis confirmed a linear inverse relationship between the SPISE index and the risk of unfavorable functional outcomes (*P* for nonlinearity = 0.83; Figure 2A). Among other surrogate indices, higher TyG index, TyG-BMI, and METS-IR were independently associated with an increased risk of unfavorable functional outcomes, whereas the TG/HDL-C ratio showed no significant association (Supplementary Table 2). Subgroup analysis demonstrated that the inverse association between the SPISE index and unfavorable functional outcomes remained consistent across most pre-specified subgroups, including different sexes, diabetes mellitus statuses, and baseline stroke severities. However, this association was far from reaching statistical significance in the subgroups of patients aged ≤ 60 years or with a BMI ≥24 kg/m^2^ (Figure 3). Regarding predictive performance comparison, ROC curve analysis indicated that METS-IR had the numerically highest discriminatory ability for predicting unfavorable functional outcomes, with an AUC of 0.580, followed by TyG-BMI (AUC = 0.576), the SPISE index (AUC = 0.557), and the TyG index (AUC = 0.555) (Figure 4). DeLong's test showed that the AUC of the SPISE index was significantly lower than those of METS-IR (*P* = 0.002) and TyG-BMI (*P* < 0.001), and comparable to that of the TyG index. The TG/HDL-C ratio (AUC = 0.505) showed no discriminatory ability for functional outcomes.

### Insulin resistance surrogates and HT

In the univariate analysis, none of the insulin resistance surrogates showed a statistically significant difference between the HT group and the non-HT group (Supplementary Table 1). However, after multivariable adjustment (Model 2), a higher TG/HDL-C ratio was independently associated with an increased risk of HT (OR 1.09, 95% CI 1.01–1.18). In contrast, the SPISE index showed no significant association with the risk of HT, either in the multivariable logistic regression model (Table 2) or in the RCS analysis (Figure 2B).

**Table 2:** Logistic regression models for identifying SPISE index and prognosis in patients with AIS.

SPISE index	Crude model		Adjusted model 1		Adjusted model 2	
	OR (95% CI)	*P*-value	OR (95% CI)	*P*-value	OR (95% CI)	*P*-value
Unfavorable functional outcomes						
Continuous variable	0.91 (0.84–0.99)	0.036	0.82 (0.73–0.91)	< 0.001	0.83 (0.74–0.92)	< 0.001
Q4:	Ref		Ref		Ref	
Q1:	1.79 (1.14–2.83)	0.013	3.19 (1.80–5.75)	< 0.001	3.08 (1.71–5.61)	< 0.001
Q2:	1.52 (0.96–2.43)	0.076	2.48 (1.41–4.43)	0.002	2.53 (1.42–4.58)	0.002
Q3:	1.20 (0.75–1.94)	0.45	1.95 (1.12–3.44)	0.019	1.97 (1.12–3.48)	0.019
*P for trend*	0.007		< 0.001		< 0.001	
Unfavorable functional outcome or HT						
Continuous variable	1.04 (0.93–1.17)	0.48	0.93 (0.81–1.06)	0.29	0.94 (0.81–1.08)	0.36
Q4	Ref		Ref		Ref	
Q1	0.87 (0.47–1.59)	0.64	1.53 (0.76–3.12)	0.23	1.51 (0.72–3.15)	0.27
Q2	0.41 (0.19–0.84)	0.018	0.61 (0.26–1.35)	0.23	0.60 (0.25–1.35)	0.23
Q3	0.70 (0.36–1.31)	0.27	0.98 (0.49–1.93)	0.95	0.95 (0.47–1.88)	0.88
*P for trend*	0.34		0.49		0.52	

Model 1 of unfavorable functional outcome was adjusted for age, sex, hypertension, atrial fibrillation, ongoing antithrombotic therapy, baseline NIHSS score, bridge therapy, baseline SBP, eGFR, HbA1c, and stroke etiology. Model 1 of HT was adjusted for age, sex, hypertension, hyperlipidemia, atrial fibrillation, current smoking, baseline NIHSS score, bridge therapy, eGFR, LDL-C, stroke etiology, and infarct location. Model 2 of unfavorable functional outcome or HT was adjusted for age, sex, hypertension, diabetes mellitus, hyperlipidemia, atrial fibrillation, coronary heart disease, history of stroke, current smoking, ongoing antithrombotic therapy, baseline NIHSS score, baseline SBP, baseline blood glucose, BMI, standard dose, OTT, bridge therapy, eGFR, HbA1c, LDL-C, stroke etiology, infarct location.

## Discussion

This study found that a lower SPISE index, a novel surrogate biomarker for insulin resistance, was independently associated with an increased risk of unfavorable 90-day functional outcomes in patients with AIS receiving intravenous thrombolysis. This association exhibited a linear dose-response relationship and remained consistent across most clinically relevant subgroups. Contrary to our initial hypothesis, however, among the five insulin resistance surrogates evaluated, the predictive ability of the SPISE index for unfavorable functional outcomes was comparable to that of the TyG index but inferior to the TyG-BMI and METS-IR. Furthermore, we observed that among these surrogate indices, only a higher TG/HDL-C ratio was associated with an increased risk of post-thrombolysis HT.

Studies have evaluated the correlation between the level of insulin resistance estimated by the aforementioned surrogate indices and that measured by the gold-standard method. Data indicate that the TyG index (r = −0.418 to −0.681) (33, 34), METS-IR (r = −0.622) (14), and SPISE index (r = 0.474 to 0.561) (23) show moderate correlations with insulin resistance measured by the gold-standard method, whereas the correlation for the TG/HDL-C ratio is weaker (r = −0.184 to −0.450) (23, 35). The relatively weaker correlation of the TG/HDL-C ratio may be attributed to the fact that it incorporates only lipid parameters, failing to account for two core components of insulin resistance, that is hyperglycemia and obesity. This difference also explains why, in the correlation analysis among the various surrogate indices, the TG/HDL-C ratio showed the weakest correlation with TyG-BMI, which incorporates both blood glucose and BMI. Although data directly assessing the correlation between TyG-BMI and the gold-standard method are lacking, existing studies suggest that TyG-BMI may be superior to the TyG index in identifying insulin resistance (AUC = 0.748 vs. 0.690) (12). Our finding that higher levels of the TyG index, TyG-BMI, and METS-IR are associated with unfavorable functional outcomes in patients with AIS after intravenous thrombolysis is consistent with prior evidence. A meta-analysis of 25 studies demonstrated that a higher TyG index, compared to a lower level, was linked to early neurological deterioration, unfavorable 3-month functional outcomes, and 1-year stroke recurrence in patients with AIS (17). Similarly, both TyG-BMI and METS-IR have been associated with early neurological deterioration and unfavorable functional outcomes in patients with AIS, regardless of reperfusion therapy status (18–20, 36, 37). However, reports on the association between the TG/HDL-C ratio and functional outcomes are conflicting, with findings ranging from a linear association (38, 39), a nonlinear association (40), to no association (41). This inconsistency likely results from heterogeneity in study populations, analytical methods, and outcome definitions, and also reflects the limited capacity of the TG/HDL-C ratio to fully capture the pathophysiology of insulin resistance.

The SPISE index has been validated as an effective tool for predicting the risk of diabetes mellitus (42), metabolic syndrome (43), cardiovascular disease (44), and stroke (45) due to its non-invasive, simple, and cost-effective nature. Our study extends the role of the SPISE index in the context of AIS from an influencing factor for incidence to a prognostic determinant. Contrary to our initial hypothesis, however, the predictive performance of the SPISE index for functional outcomes was not superior to that of TyG-BMI or METS-IR. A key distinction among these three surrogate indices lies in the inclusion of FBG. During the acute phase of AIS, patients frequently exhibit stress-induced hyperglycemia, defined as an increase in FBG of more than 20 mg/dL (46). This phenomenon is attributed to multiple mechanisms, including nonspecific autonomic, hormonal, and metabolic responses to acute stress, increased growth hormone secretion due to stroke-induced hypothalamic dysfunction, and stimulation of hypothalamic and brainstem glucoregulatory centers by local ischemia (46). Substantial evidence indicates that acute hyperglycemia is independently associated with poor neurological recovery, while insulin intervention has been shown to mitigate brain injury in animal models (22). Consequently, surrogate indices (TyG-BMI, METS-IR) that incorporate FBG are able to more sensitively capture the state of stress-induced hyperglycemia, a pathophysiological condition closely associated with unfavorable prognosis, thereby demonstrating superior predictive performance. This observation raises a deeper question: to what extent do the values of these surrogate indices (TyG, TyG-BMI, METS-IR), measured in the acute phase of AIS, reflect a dynamic worsening of insulin resistance relative to the pre-stroke state? Regrettably, there is currently no definitive answer, as no study has longitudinally assessed dynamic changes in insulin resistance using the gold-standard HEC in patients with AIS. However, supportive evidence comes from a study in critically ill patients: a comparison of HEC results between 40 critically ill patients admitted to the ICU and 25 healthy controls revealed widespread insulin resistance in the patient group, independent of disease category, baseline FBG levels, or BMI (47). This finding supports the hypothesis that acute stress can dynamically exacerbate insulin resistance. Therefore, although precise quantification is not feasible, surrogate indices that incorporate FBG may more accurately reflect the dynamic evolution of metabolic dysregulation during the acute phase of AIS.

It is important to note that the absolute AUC values of all insulin resistance surrogates were modest in our cohort. Such values indicate that these surrogate indices, when used alone, have limited ability to distinguish functional outcomes of individual patients, and are not suitable as standalone prognostic tools in clinical practice. Another limitation is that we did not evaluate whether these insulin resistance surrogates add incremental predictive value beyond established clinical predictors such as age and NIHSS score, for example through integrated discrimination improvement or net reclassification improvement metrics. Given the modest absolute discrimination of these surrogate indices observed here, their added value atop routine clinical variables is likely limited, but this remains to be formally tested in larger, multicenter cohorts.

The potential mechanisms linking insulin resistance to unfavorable functional outcomes in patients with AIS are complex and interrelated. First, metabolic disturbances associated with insulin resistance, such as hyperglycemia, hyperlipidemia, and elevated levels of blood free fatty acids (48), directly impair neuronal recovery through glucotoxicity and lipotoxicity (49). Second, a state of chronic low-grade inflammation, a hallmark of insulin resistance, partially mediates its association with adverse prognosis (50, 51). Although clinical measures primarily reflect peripheral insulin resistance, animal studies suggest a close connection between peripheral and brain insulin resistance (52). In mouse models of high-fat diet-induced brain insulin resistance, activation of microglia and astrocytes, upregulation of pro-inflammatory cytokines, and increased oxidative stress within the brain have been observed. These changes collectively impair neuroplasticity and synaptic plasticity (53–55). Third, insulin resistance-induced imbalance between vasodilatory factors (nitric oxide) and vasoconstrictive factors (endothelin-1) contributes to endothelial dysfunction (56). This provides a pathophysiological basis for the association between insulin resistance and increased cerebral small vessel disease burden (57), which is linked to insufficient salvage of the ischemic penumbra after intravenous thrombolysis and a higher risk of unfavorable functional outcomes (58, 59). Fourth, cross-sectional evidence indicates that insulin resistance is an independent predictor of poor leptomeningeal collateral circulation in patients with AIS (60). Robust collateral circulation is a critical factor in limiting final infarct volume and determining functional outcomes (61). These multifactorial mechanisms collectively support the notion that insulin resistance represents a potential therapeutic target for improving prognosis in AIS. This hypothesis has received preliminary validation from both basic and clinical research. In preclinical studies, insulin resistance-improving agents (e.g., pioglitazone, glucagon-like peptide-1 [GLP-1] receptor agonists) have demonstrated neuroprotective effects in stroke models through multiple pathways, including anti-inflammatory, antioxidant, anti-apoptotic actions, blood-brain barrier protection, and promotion of neurovascular repair (62, 63). Emerging clinical evidence also supports this concept. Liraglutide has been shown to improve 90-day functional outcomes and reduce stroke recurrence in patients with type 2 diabetes mellitus who experienced a minor stroke or transient ischemic attack (64). Furthermore, a phase 2 trial indicated that semaglutide improved 90-day functional outcomes in patients with acute large vessel occlusion undergoing endovascular treatment (65). Currently, two phase 3 trials evaluating the efficacy of semaglutide in patients with AIS receiving endovascular therapy are underway (NCT06788626; NCT07030621). Their results will provide crucial high-level evidence regarding the potential of targeting insulin resistance to improve prognosis in AIS.

Consistent with prior research, this study found no significant association between most insulin resistance surrogates of and the risk of post-thrombolysis HT (25, 26). A possible pathophysiological basis for this finding is that insulin resistance is often accompanied by a prothrombotic state characterized by hypercoagulability (66) and platelet hyperactivation (67). This state may not only reduce the risk of post-thrombolysis HT but also contribute to difficulties in achieving vascular recanalization, partially explaining the overall adverse prognosis in patients with insulin resistance. Notably, our study identified an independent association between the TG/HDL-C ratio and an increased risk of post-thrombolysis HT. Given that this ratio exhibits the weakest correlation with other surrogate indices and comprises only lipid parameters, this association may plausibly reflect direct effects of lipid metabolism on blood–brain barrier integrity, rather than insulin resistance *per se*. Preclinical evidence supports this hypothesis: exogenous HDL administration has been shown to reduce blood–brain barrier permeability and attenuate HT in rodent stroke models (68). However, our study was not designed to test this mechanism directly, and we cannot exclude residual confounding by unmeasured lipid-related pathways. Further basic and clinical studies are required to validate this potential link.

This study has several limitations. First, there are selection bias in patient recruitment. The enrolled cohort exhibited overall mild neurological deficits, with a median NIHSS score of four, because critically ill patients were often excluded due to their inability to accurately report height and weight for BMI calculation, typically a result of impaired consciousness, aphasia, or bedridden status. Our findings may not generalize to patients with severe acute ischemic stroke, who were underrepresented in our cohort. Future studies enrolling broader severity spectra are needed to validate these associations. Second, insulin resistance surrogates were calculated from a single fasting blood sample collected on the morning after admission. Metabolic status, including stress-induced hyperglycemia and lipid fluctuations, evolves dynamically in the acute phase of AIS. A single time point measurement approach may underestimate the true association between insulin resistance and outcomes. Consistent with recent evidence emphasizing the importance of dynamic metabolic metrics in patients undergoing craniotomy (69), future studies using serial sampling to assess dynamic changes, cumulative burden, or delta values are needed to address this gap. Third, although we employed multivariable logistic regression models to adjust for known confounders, the retrospective design of this study precluded the assessment of several potentially important confounding variables closely related to insulin resistance, such as dietary patterns, physical activity levels, and sleep quality (9). Lastly, the generalizability of our findings is limited. Similar to most studies in this field, our data were derived from a hospitalized Chinese population. Therefore, caution is warranted when extrapolating the conclusions to other ethnicities, geographical regions, and populations with different lifestyles. In light of these limitations, future studies are warranted to validate our findings in populations of diverse ethnicities, regions, and stroke severity to assess their broader applicability. Additionally, prospective studies that dynamically monitor insulin resistance surrogates at multiple time points and collect more comprehensive lifestyle and clinical data would help to more accurately elucidate the complex relationship between insulin resistance and prognosis in AIS.

## Conclusion

This study confirms that elevated levels of insulin resistance surrogates, specifically the SPISE index, TyG index, TyG-BMI, and METS-IR, are independently associated with an increased risk of unfavorable 90-day functional outcomes following intravenous thrombolysis in patients with AIS. These findings underscore the importance of insulin resistance in AIS prognosis, but highlight that their standalone discriminative utility remains limited. In this study, insulin resistance surrogates showed no significant association with post-thrombolysis HT. An important exception was the TG/HDL-C ratio, which was independently associated with an increased risk of post-thrombolysis HT. This association likely reflects a direct effect of lipid metabolism on blood–brain barrier integrity rather than being mediated through insulin resistance. Given that pharmacological agents targeting insulin resistance, particularly GLP-1 receptor agonists, have shown neuroprotective potential in preclinical and preliminary clinical studies, large-scale randomized controlled trials aimed at mitigating insulin resistance in patients with AIS are needed to establish high-level evidence? for guiding acute-phase therapeutic strategies.

## Data Availability

The raw data supporting the conclusions of this article will be made available by the authors, without undue reservation.
