# Supplementary material for: Association of the single point insulin sensitivity estimator index with functional outcomes and hemorrhagic transformation in patients with acute ischemic stroke
Source: Front Nutr. 2026 Jun 22;13:1805357. doi: 10.3389/fnut.2026.1805357 (PMC13334846; doi:10.3389/fnut.2026.1805357)
Supplement: Supplementary file 1 [file Supplementary_file_1.docx]

**Association of the Single Point Insulin Sensitivity Estimator Index with Functional Outcomes and Hemorrhagic Transformation in Patients with Acute Ischemic Stroke**

**Table S1. Characteristics of patients stratified by HT**

**Table S2. Logistic regression models for identifying insulin resistance surrogates index and prognosis in patients with AIS**

**Figure S1. Patient selection flowchart**

**Table S1. Characteristics of patients stratified by HT**

| **Characteristic** | **Non-HT (N = 786)** | **HT (N = 76)** | ***P*-value** |
| --- | --- | --- | --- |
| Age, years, mean ± SD | 67.5 ± 13.2 | 74.5 ± 11.4 | <0.001 |
| Sex, n (%) |  |  | 0.027 |
| Female | 244 (31.0) | 33 (43.4) |  |
| Male | 542 (69.0) | 43 (56.6) |  |
| Hypertension, n (%) | 614 (78.1) | 67 (88.2) | 0.040 |
| Diabetes mellitus, n (%) | 208 (26.5) | 20 (26.3) | 0.98 |
| Hyperlipidemia, n (%) | 313 (39.8) | 22 (28.9) | 0.063 |
| Atrial fibrillation, n (%) | 167 (21.2) | 39 (51.3) | <0.001 |
| Coronary heart disease, n (%) | 50 (6.4) | 6 (7.9) | 0.62 |
| History of stroke, n (%) | 130 (16.5) | 14 (18.4) | 0.67 |
| Current smoking, n (%) | 272 (34.6) | 16 (21.1) | 0.017 |
| Ongoing antithrombotic therapy,  n (%) | 115 (14.6) | 13 (17.1) | 0.56 |
| Baseline NIHSS score,  median (Q1 − Q3) | 4.0 (2.0 − 8.0) | 9.0 (4.0 − 14.5) | <0.001 |
| Baseline SBP, mmHg,  mean ± SD | 153.8 ± 22.3 | 152.5 ± 21.1 | 0.61 |
| Baseline DBP, mmHg,  mean ± SD | 86.5 ± 15.0 | 85.9 ± 14.0 | 0.71 |
| BMI, kg/m^2^, mean ± SD | 23.6 ± 3.7 | 23.3 ± 3.4 | 0.45 |
| Thrombolytic drug,  n (%) |  |  | 0.61 |
| Alteplase | 775 (98.6) | 76 (100.0) |  |
| Tenecteplase | 11 (1.4) | 0 (0.0) |  |
| Standard dose,  n (%) | 717 (91.2) | 70 (92.1) | 0.79 |
| OTT, min, median (Q1 − Q3) | 150.0 (110.0 − 210.0) | 160.0 (110.0 – 205.5) | 0.85 |
| Bridge therapy, n (%) | 33 (4.2) | 13 (17.1) | <0.001 |
| eGFR, mL/min/1.73m², median (Q1 − Q3) | 90.6 (72.1 − 100.6) | 84.4 (59.5 − 94.2) | <0.001 |
| FPG, mg/dL, median (Q1 − Q3) | 94 (83 − 112) | 101 (90 − 125) | 0.011 |
| HbA1c, %, median (Q1 − Q3) | 5.8 (5.5 − 6.4) | 5.8 (5.6 − 6.5) | 0.62 |
| TC, mg/dL, mean ± SD | 171.9 ± 42.6 | 160.5 ± 36.8 | 0.013 |
| LDL-C, mg/dL, mean ± SD | 107.1 ± 33.6 | 95.3 ± 29.9 | 0.002 |
| HDL-C, mg/dL, mean ± SD | 44.0 ± 10.9 | 43.6 ± 12.6 | 0.83 |
| TG, mg/dL, median (Q1 − Q3) | 115.1 (85.0 − 163.9) | 97.9 (72.2 − 163.0) | 0.069 |
| Stroke etiology, n (%) |  |  | <0.001 |
| Large artery atherosclerosis | 178 (22.6) | 16 (21.1) |  |
| Cardioembolism | 172 (21.9) | 33 (43.4) |  |
| Small vessel occlusion | 242 (30.8) | 6 (7.9) |  |
| Other determined or undetermined | 194 (24.7) | 21 (27.6) |  |
| Infarct location, n (%) |  |  | 0.078 |
| Anterior circulation | 577 (73.4) | 64 (84.2) |  |
| Posterior circulation | 176 (22.4) | 9 (11.8) |  |
| Anterior and posterior circulation | 33 (4.2) | 3 (3.9) |  |
| SPISE index, median (Q1 − Q3) | 6.8 (5.6 − 8.1) | 7.1 (5.5 − 8.5) | 0.47 |
| Quartiles of SPISE index, n (%) |  |  | 0.10 |
| Q1 | 194 (24.7) | 22 (28.9) |  |
| Q2 | 204 (26.0) | 11 (14.5) |  |
| Q3 | 197 (25.1) | 18 (23.7) |  |
| Q4 | 191 (24.3) | 25 (32.9) |  |
| TyG index, median (Q1 − Q3) | 8.6 (8.3 − 9.1) | 8.7 (8.2 − 9.1) | 0.59 |
| TyG-BMI, mean ± SD | 206.7 ± 39.6 | 204.1 ± 37.1 | 0.56 |
| TG/HDL-C ratio, median (Q1 − Q3) | 2.7 (1.9 − 4.2) | 2.5 (1.5 − 4.1) | 0.22 |
| METS-IR, median (Q1 − Q3) | 36.1 (31.8 – 41.3) | 35.6 (32.3 − 42.1) | 0.87 |

BMI, body mass index; DBP, diastolic blood pressure; eGFR, glomerular filtration rate; FPG, fasting plasma glucose; HbA1c, Hemoglobin A1c; HDL-C, high-density lipoprotein cholesterol; HT, hemorrhagic transformation; LDL-C, low-density lipoprotein cholesterol; METS-IR, metabolic score for insulin resistance; NIHSS, National Institutes of Health Stroke Scale; OTT, onset-to-treatment time; SBP, systolic blood pressure; SD, standard deviation; SPISE, single point insulin sensitivity estimator; TC, total cholesterol; TG, triglyceride; TyG, triglyceride-glucose.

**Table S2. Logistic regression models for identifying insulin resistance surrogates index and prognosis in patients with AIS**

|  | **Insulin resistance surrogates** | **Crude Model** | | **Adjusted Model 1** | | **Adjusted Model 2** | |
| --- | --- | --- | --- | --- | --- | --- | --- |
|  |  | **OR** (**95% CI**) | ***P*-value** | **OR** (**95% CI**) | ***P*-value** | **OR** (**95% CI**) | ***P*-value** |
| Unfavorable functional outcome | TyG index | 1.30 (1.02 – 1.65) | 0.030 | 1.75 (1.24 − 2.49) | 0.002 | 1.71 (1.19 − 2.48) | 0.004 |
|  | TyG-BMI | 1.01 (1.00 − 1.01) | 0.001 | 1.01 (1.01 − 1.02) | <0.001 | 1.01 (1.00 − 1.02) | <0.001 |
|  | TG/HDL-C ratio | 1.02 (0.97 − 1.06) | 0.50 | 1.04 (0.98 − 1.10) | 0.24 | 1.03 (0.97 − 1.09) | 0.42 |
|  | METS-IR | 1.04 (1.01 − 1.06) | 0.001 | 1.06 (1.03 − 1.09) | <0.001 | 1.05 (1.02 − 1.08) | <0.001 |
| HT | TyG index | 1.05 (0.72 − 1.49) | 0.80 | 1.57 (1.02 − 2.41) | 0.037 | 1.64 (0.98 − 2.72) | 0.055 |
|  | TyG-BMI | 1.00 (0.99 − 1.00) | 0.58 | 1.00 (1.00 − 1.01) | 0.37 | 1.00 (1.00 − 1.01) | 0.45 |
|  | TG/HDL-C ratio | 1.04 (0.98 − 1.10) | 0.11 | 1.10 (1.02 − 1.18) | 0.022 | 1.09 (1.01 − 1.18) | 0.034 |
|  | METS-IR | 1.00 (0.97 − 1.03) | 0.98 | 1.02 (0.98 − 1.05) | 0.29 | 1.02 (0.98 − 1.06) | 0.32 |

Model 1 of unfavorable functional outcome was adjusted for age, sex, hypertension, atrial fibrillation, ongoing antithrombotic therapy, baseline NIHSS score, bridge therapy, baseline SBP, eGFR, HbA1c, and stroke etiology. Model 1 of HT was adjusted for age, sex, hypertension, hyperlipidemia, atrial fibrillation, current smoking, baseline NIHSS score, bridge therapy, eGFR, LDL-C, stroke etiology, and infarct location. Model 2 of unfavorable functional outcome or HT was adjusted for age, sex, hypertension, diabetes mellitus, hyperlipidemia, atrial fibrillation, coronary heart disease, history of stroke, current smoking, ongoing antithrombotic therapy, baseline NIHSS score, baseline SBP, baseline blood glucose, BMI, standard dose, OTT, bridge therapy, eGFR, HbA1c, LDL-C, stroke etiology, infarct location.


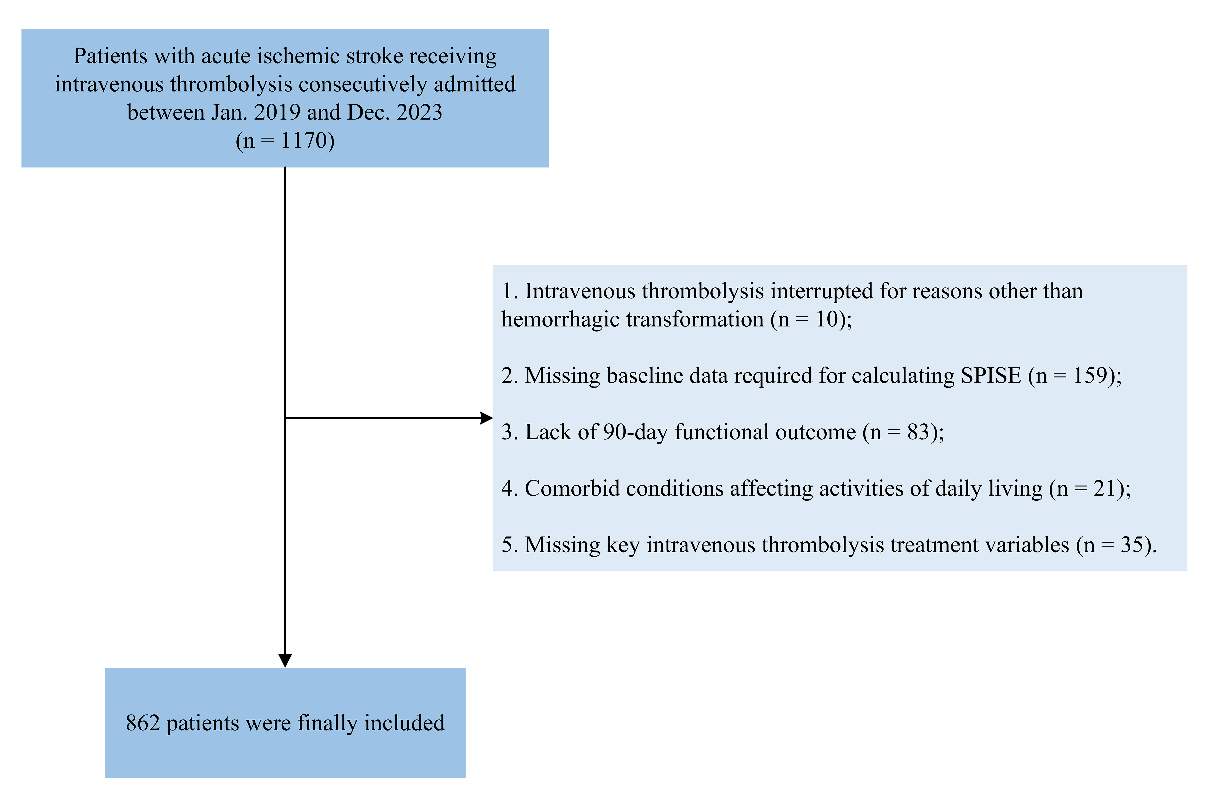


**Figure S1. Patient selection flowchart**
